# Conversion of epithelial-to-mesenchymal transition to mesenchymal-to-epithelial transition is mediated by oxygen concentration in pancreatic cancer cells

**DOI:** 10.3892/ol.2022.13227

**Published:** 2022-02-04

**Authors:** Shuo Chen, Xi Chen, Wei Li, Tao Shan, Wan Run Lin, Jiancang Ma, Xijuan Cui, Wenbin Yang, Gang Cao, Yiming Li, Li Wang, Ya'an Kang

Oncol Lett 15: 7144-7152, 2018; DOI: 10.3892/ol.2018.8219

Subsequently to the publication of the above paper, an interested reader drew to the authors’ attention that a number of apparent anomalies existed with certain of the data presented in one of figures. Specifically, there appeared to be a pair of cells within the cellular images shown in the data panel of Fig. 2B (portraying the vimentin/hypoxia experiment) that were matching on p. 7147. Furthermore, adjusting the contrast settings of the figure revealed the possibility that certain of the cells were not intrinsic to the original photographs taken of the presented data.

Following an internal enquiry, the Editor of *Oncology Letters* shares the same concerns regarding the authenticity of these images that were raised by the interested reader; therefore, in view of the potential anomalies that have been identified and owing to a lack of overall confidence in the presented data, the Editor has decided to retract the above paper from the publication. After having passed on this decision to the authors, they were in agreement that the paper should be retracted. The Editor apologizes to the readership of the Journal for any inconvenience caused.

